# CD47 in Osteosarcoma: Correlation with Metastasis and Macrophage-Mediated Phagocytosis

**DOI:** 10.3390/cells13221862

**Published:** 2024-11-10

**Authors:** Yunmi Ko, Seog-Yun Park, Jong Woong Park, June Hyuk Kim, Hyun Guy Kang, Jun Ah Lee

**Affiliations:** 1 Department of Pediatrics, National Cancer Center, Goyang 10408, Republic of Korea; 75302@ncc.re.kr; 2 Department of Pathology, National Cancer Center, Goyang 10408, Republic of Korea; 11740@ncc.re.kr; 3 Orthopedic Oncology Clinic, Center for Rare Cancer, National Cancer Center, Goyang 10408, Republic of Korea; jwpark82@ncc.re.kr (J.W.P.); docjune@ncc.re.kr (J.H.K.); ostumor@ncc.re.kr (H.G.K.)

**Keywords:** osteosarcoma, CD47, metastasis, macrophage, immunotherapy

## Abstract

CD47 is expressed on cell surfaces and acts as a “don’t eat me” signal by interacting with signal-regulatory protein-α on the macrophage surface. Some cancer cells express CD47 protein and can evade macrophage phagocytosis. Herein, we evaluated the feasibility of targeting CD47 for osteosarcoma by analyzing its expression patterns, clinicopathological correlations, and immunotherapeutic potential. We performed a retrospective analysis on 24 biopsy samples from patients with osteosarcoma to investigate correlations between CD47 protein positivity and clinicopathological characteristics. CD47 protein expression was detected in 20.8% of the biopsy samples. CD47 positivity correlated with metastasis at diagnosis. Patients with CD47-positive tumors were older than those with CD47-negative tumors. However, CD47 protein expression was not associated with sex, tumor size, or histologic response to preoperative chemotherapy. In vitro, CD47 antibody (B6H12) did not affect osteosarcoma cell viability or apoptosis. In a wound-healing assay, CD47 inhibited the migration of osteosarcoma cells. Differentiated macrophages exhibited higher phagocytic activity against osteosarcoma cells when pretreated with B6H12 compared with the isotype control. Our preliminary data suggest a possible interaction between CD47 protein and macrophage phagocytosis in osteosarcoma metastasis. A better understanding of the role of CD47 is necessary to develop an innovative immunotherapeutic approach against osteosarcoma.

## 1. Introduction

Osteosarcoma, an immunogenic tumor, is a focal point of diverse immunotherapies [1,2,3]. However, it lacks specific target antigens, leading to nonspecific immune responses in clinical trials utilizing muramyl tripeptide-phosphatidyl ethanolamine and interferons [4,5,6]. Immune checkpoint inhibitors targeting programmed cell death protein 1/programmed death-ligand 1 and cytotoxic T-lymphocyte-associated protein 4 (CTLA-4) improve the survival of patients with refractory cancer [7,8,9,10]. Although some patients with undifferentiated pleomorphic sarcomas and liposarcomas show positive responses to these inhibitors, most patients with sarcomas do not exhibit any objective response to immune checkpoint inhibitors [11,12,13]. Insufficient T-cell responses and limited tumor-specific cytotoxic T-cells in the immunosuppressive tumor microenvironment are often considered the primary reasons for suboptimal responses [14,15]. The tumor microenvironment comprises diverse cell populations, including lymphocytes, fibroblasts, and macrophages [16,17]. A comprehensive analysis of 26 sarcoma types revealed macrophages as the predominant cell population infiltrating tumor tissues [18]. The INT-0133 clinical trial demonstrated the efficacy of muramyl tripeptide-phosphatidyl ethanolamine in enhancing the survival of patients with osteosarcoma [19]. These findings suggest the potential therapeutic value of immunotherapies targeting macrophage phagocytosis in sarcomas.

CD47 functions as a “don’t eat me” signal by interacting with signal-regulatory protein-α (SIRPα) on macrophages and affects various cellular processes, including cell proliferation, apoptosis, and immune responses [20,21,22,23]. Elevated expression of the CD47 protein in cancer cells provides a mechanism for immune evasion, particularly against phagocytosis. The CD47 protein functions as a negative regulator of the cytotoxic T-cell response against cancer cells, potentially contributing to tumor progression. Clinical investigations have revealed an association between high CD47 expression in ovarian and gastric cancer cells and unfavorable prognosis [24,25]. Despite active preclinical and clinical evaluations of recently developed CD47 antibodies, especially in hematological malignancies, data on sarcomas remain limited.

This study aimed to evaluate the feasibility of CD47 as a potential therapeutic target for osteosarcoma. Our primary aim was to delineate the expression profile of CD47 in osteosarcoma samples and investigate correlations between CD47 protein expression and various clinicopathological characteristics. Furthermore, we examined the effect of CD47 on the cell viability, apoptosis, migration, and phagocytosis of osteosarcoma cells, exploring its potential as a candidate for osteosarcoma immunotherapy.

## 2. Materials and Methods

### 2.1. CD47 Expression in Osteosarcoma Samples

A retrospective analysis was conducted on 24 patients with primary osteosarcoma treated at the National Cancer Center Hospital between 2003 and 2021. This study was approved by the Institutional Review Board of the National Cancer Center Hospital (IRB No. NCC2022-0046). Patients meeting the following criteria were included: (1) age < 20 years at the time of diagnosis, (2) high-grade osteosarcoma, (3) tumors located in the extremities, (4) no history of previous treatment, and (5) availability of the incisional biopsy specimen obtained at the time of diagnosis. Patient attributes are summarized in Table 1. Representative areas were selected from the incisional biopsy samples and visualized on a Zymed non-biotin amplification system (Zymed Laboratories, San Francisco, CA, USA). Immunohistochemical staining was performed using allophycocyanin (APC)-conjugated anti-human CD47 (B6H12; Thermo Fisher Scientific, Waltham, MA, USA) and APC-conjugated mouse IgG1κ isotype control (MOPC-21; BioLegend, San Diego, CA, USA) antibodies. Staining intensity was interpreted as follows: (1) low, CD47 positivity of <10% of osteosarcoma cells; (2) intermediate, positivity of 10%–50% of osteosarcoma cells; and (3) high, positivity of >50% of tumor cells. Osteosarcoma samples with high to intermediate staining were considered positive for CD47.

### 2.2. Cell Viability and Apoptosis Assay

Human osteosarcoma cell lines, KHOS/NP, MG-63, and U-2 OS, were obtained from the American Type Culture Collection (Manassas, VA, USA) and cultured in complete α-MEM supplemented with 10% fetal bovine serum (both from Thermo Fisher Scientific) at 37 °C in a humidified incubator with 5% CO_2_. These cells were treated with either the isotype control or anti-CD47 (B6H12; Bio X Cell, Lebanon, NH, USA) antibody for 24, 48, and 72 h across various concentrations. Cell viability was evaluated using the EZ-CYTOX assay kit (DoGenBio, Cheongju, Republic of Korea), and apoptosis with the Annexin V Detection kit (Thermo Fisher Scientific) by fluorescence-activated cell sorting (FACS), following the manufacturer’s instructions for both assays.

### 2.3. Wound-Healing Assay

MG-63 cells were cultured to 70%–80% confluence and scratched using a yellow pipette tip. The cells were subsequently treated with either isotype control or anti-CD47 (B6H12; Bio X Cell) antibody in basal media at a final concentration of 10 μg/mL for 24 and 48 h. Wound closure was monitored using an optical microscope and quantified using ImageJ software (National Institutes of Health, Bethesda, MD, USA) using the wound healing size tool plugin [26].

### 2.4. Flow Cytometry Analysis

Human CD47 (B6H12) and isotype control antibodies were purchased from Bio X Cell. For FACS analysis, the cells were stained with anti-human CD47, CD3, CD11b, and CD14 antibodies, as well as an isotype control, and analyzed using BD FACSVerse (all from BD Biosciences, San Jose, CA, USA). Specifically, 10,000–100,000 events were acquired per sample and analyzed using FACSuite and FlowJo software (both from BD Biosciences).

### 2.5. Macrophage Differentiation from Peripheral Blood

After obtaining informed consent, 40–50 mL of fresh peripheral blood was obtained from healthy volunteers, and peripheral blood mononuclear cells (PBMCs) were isolated by Lymphoprep gradient centrifugation (StemCell Technologies, Vancouver, BC, Canada). Subsequently, human CD14 microbeads (Miltenyi Biotec Inc., Bergisch Gladbach. Germany) were used to isolate monocytes from PBMCs following the manufacturer’s instructions. The isolated CD14-positive monocytes were cultured in macrophage media with recombinant human macrophage colony-stimulating factor (*rh* M-CSF; all from StemCell Technologies) for 6 d to facilitate their differentiation into macrophages. The efficiency of monocyte differentiation into macrophages was evaluated by flow cytometry using fluorescently labeled CD14 and CD11b antibodies (CD14-high and CD11b-high macrophages).

### 2.6. In Vitro Macrophage-Mediated Phagocytosis Assay

KHOS/NP osteosarcoma cells were labeled with CellTrace™ CFSE or Violet (both from Thermo Fisher Scientific) following the manufacturer’s protocol for live-cell tracking. Subsequently, the cells were stimulated with anti-CD47 or isotype control antibodies for 30 min to block the CD47 protein on the cell surface before adding monocyte-derived macrophages (MDMs) at half the ratio of the osteosarcoma cells. After 2 h of incubation at 37 °C, the percentage of macrophage-mediated phagocytosis was determined by flow cytometry using fluorescently labeled CD14 and CD11b antibodies (CellTrace-high, CD14-high, and CD11b-high) [27].

### 2.7. Statistical Analysis

Variables included age, sex, tumor size, presence of metastasis at the time of diagnosis, and the histological response to preoperative chemotherapy. The chi-square test was used to analyze the correlation between categorical clinical variables and CD47 protein expression. SPSS ver. 15.0 (SPSS Inc., Chicago, IL, USA) was used for all calculations, and statistical significance was set at *p* < 0.05. All in vitro experiments were independently conducted in triplicate at a minimum. Statistical analyses were performed using GraphPad Prism software (GraphPad Software Inc., San Diego, CA, USA) and the significance levels were * *p* < 0.05, ** *p* < 0.01, and *** *p* < 0.001.

## 3. Results

### 3.1. CD47 Expression and Clinical Characteristics

**Figure 1:**
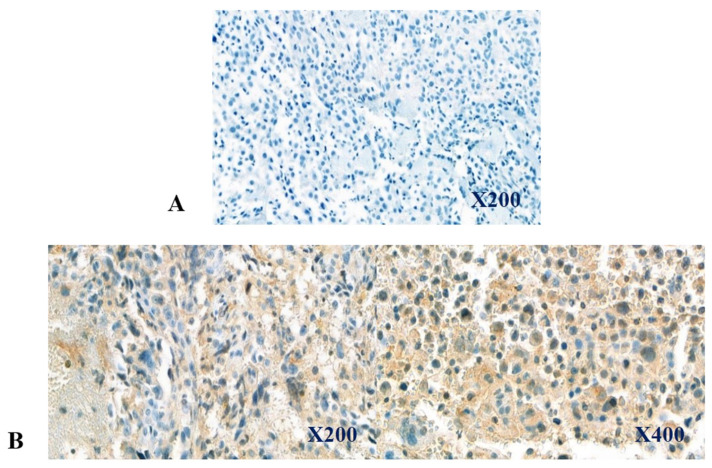
Immunohistochemical expression of CD47 in biopsy samples. The depicted regions were selected from incisional biopsy samples, and immunohistochemical staining was performed as described under Materials and Methods. The upper panel (A, from case 14) depicts CD47-negative expression, while the lower panel (B, from case 15) depicts CD47-positive expression, showing CD47-positive cells stained in light brown color.

Of the 24 osteosarcoma biopsy samples, 5 (20.8%) exhibited positive staining for CD47, with staining intensities categorized as high and low in 2 and 3 samples, respectively (Table 1, Figure 1). CD47 protein expression was associated with metastasis at diagnosis (*p* = 0.04; Table 2). Patients with tumors positive for CD47 tended to be older than those with negative staining (*p* = 0.07). However, CD47 staining was not significantly associated with sex, tumor size, or the histological response to preoperative chemotherapy (Table 2).

**Table 1:** Characteristics of the 24 patients treated at the National Cancer Center Hospital between 2003 and 2021.

Case No.	Sex/Age (y)	Tumor Location	Tumor Size (cm)	Meta at Dx/Site	TNR	CD47+	Event/Time (mo)	FU (mo)	Current Status
1	F/10.2	Distal femur	6.5 × 5.5 × 3	−	97%			165	CDF
2	M/13.0	Humerus shaft	16	−	40%			157	CDF
3	F/14.0	Proximal humerus	4.5 × 4.5 × 3.0	−	95%			143	CDF
4	M/18.8	Distal femur	11.4 × 8.4	−	95%			123	CDF
5	M/16.3	Proximal tibia	4.2 × 1.0 × 1.0	+/lung	95%		lung meta/14.1	33	DOD
6	M/14.7	Distal femur	11.8 × 8.0 × 3.9	−	100%			115	CDF
7	F/16.1	Distal femur	6.3 × 5.0 × 4.5	−	95%			107	CDF
8	M/13.9	Distal femur	9.0 × 3.1 × 3.0	+/bone	99%		lung meta/25.6	63	DOD
9	M/17.7	Proximal tibia	5.2 × 3.0 × 2.6	−	95%	+	lung meta/16.8	57	DOD
10	M/15.9	Distal tibia	6.0 × 4.0 × 3.7	−	99%			94	CDF
11	F/17.6	Distal radius	4.4 × 2.6 × 2.3	−	95%		local recur/18.0	98	NED
12	M/17.7	Distal femur	10.5 × 5.2 × 5.0	−	70%			71	CDF
13	M/24.5	Distal femur	13.3 × 5.2 × 3.8	−	60%	+	lung meta/16.7 bon meta/41	66	DOD
14	M/20.3	Humerus shaft	23	+/lung	95%			67	CDF
15	M/18.3	Humerus shaft	5.7 × 7.2 × 10	+/lung	100%	+		51	CDF
16	M/17.4	Proximal tibia	2.6 × 3.6 × 4.6		100%			47	CDF
17	F/12.4	Proximal tibia	4.8 × 6.6 × 5.8	−	40%		lung meta/4.5	44	AWD
18	F/14	Proximal tibia	3.7 × 4.1 × 6.9	−	70%	+		44	CDF
19	M/21.9	Proximal fibula	4.9 × 5.1 × 9.0	−	80%		local recur/15.5	41	NED
20	F/11.0	Distal femur	11.8	−	95%			40	CDF
21	M/15.1	Distal femur	5.2 × 7.6 × 5.6	−	95%			30	CDF
22	M/15.7	Distal femur	21.6	−	10%		lung meta/12	31	NED
23	M/15.9	Proximal tibia	15.3 × 6.9 × 6.4	+/lung, bone	60%		lung meta/11 bone meta/14	27	DOD
24	F/7.8	Distal femur	13	+/lung	50%	+	lung meta/7	17	DOD

DOD, died of disease; CDF, continuously disease-free; NED, no evidence of disease; AWD, alive with the disease; TNR, tumor necrosis rate; FU, follow up.

### 3.2. Effect of CD47 Antibody on Osteosarcoma Cells

**Figure 2:**
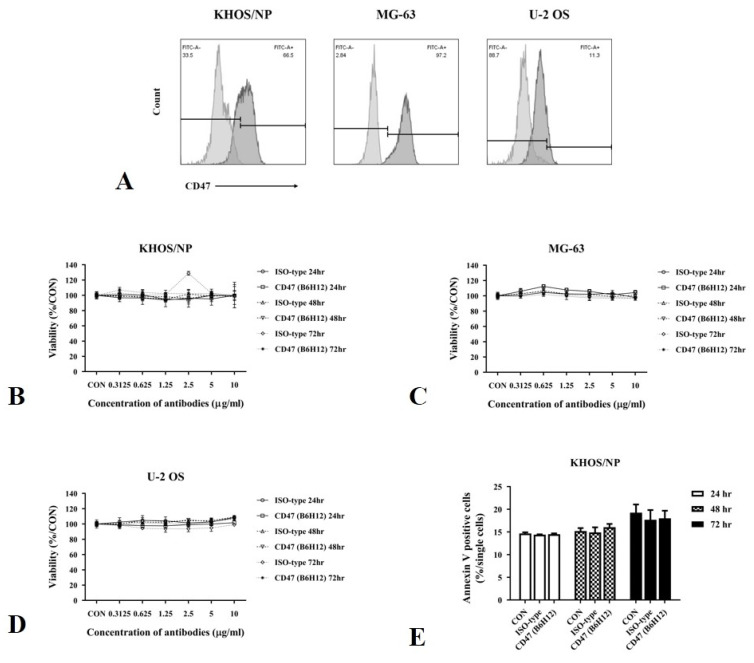
In vitro efficacy of CD47 antibody in osteosarcoma cells. (**A**) The expression of CD47 is analyzed using FACS analysis in osteosarcoma cells. Representative images display the histogram of CD47 expression under basal conditions. (**B**–**D**) Osteosarcoma cells are treated with either isotype control or anti-CD47 (B6H12) antibody at the indicated concentrations for 24, 48, and 72 h. The effect of the anti-CD47 antibody on osteosarcoma cell viability is assessed using the EZ-CyTox cell viability assay kit. (**E**) KHOS/NP osteosarcoma cells are exposed to an isotype control or anti-CD47 (B6H12) antibody at the indicated concentrations for 24, 48, and 72 h. The effect of the anti-CD47 antibody on KHOS/NP cell apoptosis is evaluated using the Annexin V Detection kit.

We examined the protein expression of CD47 in KHOS/NP, MG-63, and U-2 OS cells under basal culture conditions. KHOS/NP, MG-63, and U-2 OS cells were treated with the CD47 antibody (B6H12) to assess the effect of the antibody on osteosarcoma cells. However, the B6H12 antibody did not demonstrate dose-dependent inhibitory effects on osteosarcoma cell viability (Figure 2A–C). Similarly, B6H12 exerted no substantial effect on apoptosis of KHOS/NP cells (Figure 2D). 

**Figure 3:**
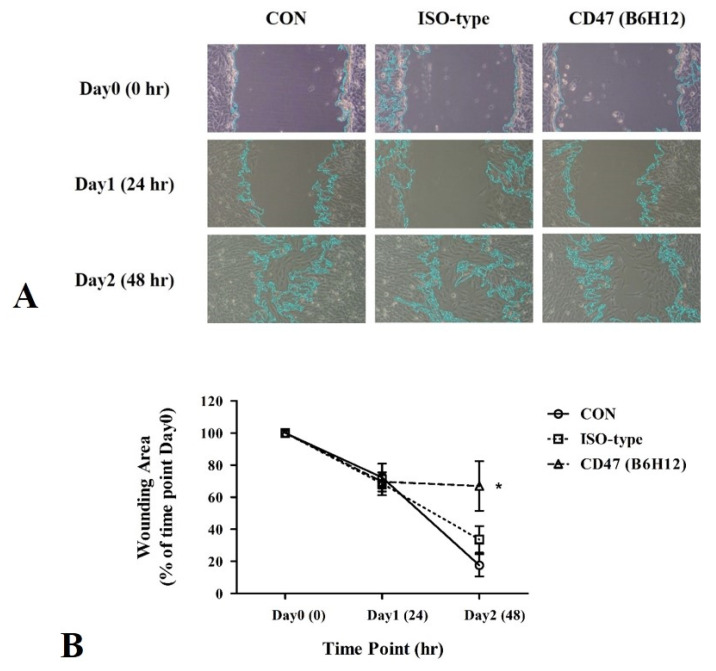
Wound-healing assay to assess migration of osteosarcoma cells following CD47 (B6H12) antibody treatment. (**A**) MG-63 osteosarcoma cells are treated with a 10 μg/mL concentration of an isotype control antibody or the anti-CD47 antibody (B6H12). Representative images show the area the cells cover at the indicated time points after in vitro scratch wounding. (**B**) The summary graph illustrates the percentage of wound closure in MG-63 osteosarcoma cells over time compared with untreated controls. Statistical analysis of wound closure is conducted using ImageJ software with the wound healing size plug-in. The percentages are represented as the mean ± standard error of the mean, and statistical significance is determined by comparison with the control (* *p* < 0.05).

**Figure 4:**
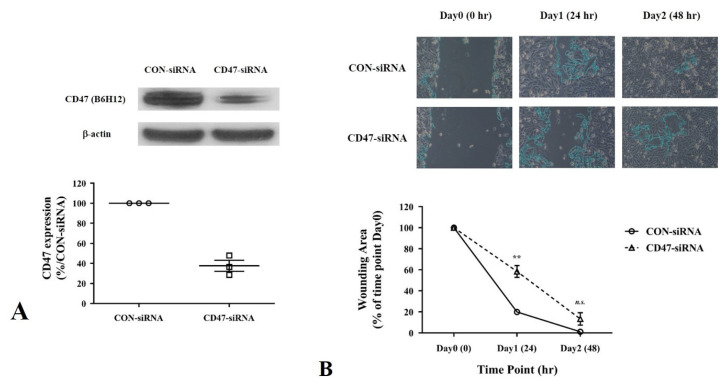
Wound-healing assay to assess migration of osteosarcoma cells following CD47 siRNA transfection. (**A**) MG-63 osteosarcoma cells are treated with either control siRNA or CD47 siRNA, and Western blot analysis is performed to evaluate CD47 expression. The summary graph illustrates the CD47 expression levels in the MG-63 osteosarcoma cells. (**B**) Representative images display the area covered by MG-63 osteosarcoma cells at the indicated time points following in vitro scratch wounding. The summary graph illustrates the percentage of wound closure in MG-63 cells over time compared to the control siRNA. Statistical analysis of wound closure is performed using ImageJ software with the wound healing size plug-in. The percentages are represented as the mean ± standard error of the mean, and statistical significance is determined by comparison with the control siRNA (** *p* < 0.01, *n.s.*: no significance).

In the retrospective analysis, a correlation was found between CD47 protein expression and metastasis at the time of diagnosis. A subsequent experiment was conducted using MG-63 cells to validate this finding. MG-63 cells were treated with B6H12 and subjected to a wound-healing assay. B6H12 suppressed the migration of MG-63 cells compared with the isotype antibody (Figure 3A,B). Furthermore, CD47 silencing also appeared to interfere with the migration of MG-63 cells (Figure 4A,B).

### 3.3. Effect of CD47 Antibody on the Phagocytic Activity of MDMs Toward Osteosarcoma Cells

**Figure 5:**
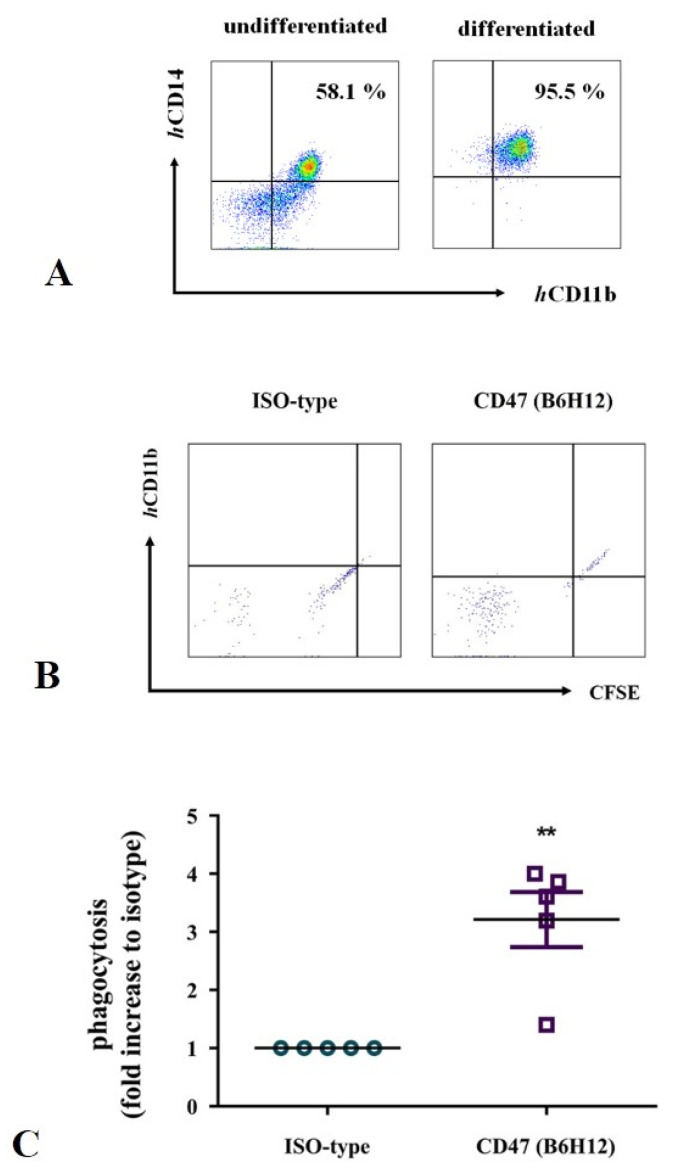
Phenotypic characterization of monocyte-derived macrophages and comparative analysis of in vitro macrophage-mediated phagocytosis following CD47 (B6-H12) antibody treatment. (**A**) CD14-positive monocytes are isolated and cultured in a macrophage medium, followed by activation with recombinant human M-CSF for 6 d. Single cells are stained and analyzed using the BD FACSVerse System. (**B**,**C**) KHOS/NP cells are labeled with CellTrace™ CFSE dye (KHOS-CFSE) and incubated with 10 μg/mL anti-CD47 antibody or IgG ISO-type antibody for 30 min at 37 °C. KHOS/NP-CFSE cells are co-cultured with differentiated macrophages for 2 h. The percentage of macrophage-mediated phagocytosis (CD14-high, CellTrace-high, and CD11b-high) is measured by flow cytometry. Data are recorded using BD FACSVerse and analyzed using FlowJo software. The percentages are presented as the mean ± standard error of the mean, and statistical significance is determined by comparison with the IgG ISO-type antibody (** *p* < 0.01).

CD14-positive monocytes were isolated from PBMCs of healthy donors to establish a streamlined protocol for MDM generation. The efficacy of the differentiation of CD14-positive monocytes, which was achieved following exposure to *rh* M-CSF, was evaluated by FACS. Consequently, approximately 97% of the viable cells exhibited macrophage phenotypes (CD14-high and CD11b-high; Figure 5A), confirming the effectiveness of our protocol.

KHOS/NP osteosarcoma cells were labeled with carboxyfluorescein succinimidyl ester and pretreated with B6H12 antibody to assess the potential enhancement of macrophage-mediated phagocytosis in osteosarcoma cells mediated by the CD47 antibody. Phagocytic activity was evaluated by FACS following the co-culture of the generated MDMs. The phagocytic activity of MDMs on KHOS/NP osteosarcoma cells was higher in cells pretreated with the CD47 antibody, exhibiting a 3.2-fold increase compared with those pretreated with the isotype antibody (*p* = 0.0016; Figure 5B,C). These findings demonstrate that the CD47 antibody enhances the phagocytic activity of MDMs toward osteosarcoma cells.

## 4. Discussion

This study evaluated the feasibility of using CD47 as a potential therapeutic target for osteosarcoma and investigated the correlation between CD47 expression and clinicopathological characteristics using biopsy samples obtained from patients with osteosarcoma. Our findings indicated that positive staining for the CD47 protein correlated with metastasis at diagnosis and patient age. Conversely, no significant associations were observed with sex, tumor size, or the histological response to preoperative chemotherapy. We evaluated the potential of CD47 as an immunotherapy target in osteosarcoma cells. Our results demonstrated that the CD47 antibody and silencing with siRNA could suppress the migration of osteosarcoma cells, which may be used in treating metastatic osteosarcoma. We successfully generated MDMs from human PBMCs using a streamlined protocol. The CD47 antibody enhanced the phagocytic activity of MDMs towards osteosarcoma cells, further supporting its potential value in immunotherapeutic approaches for osteosarcoma.

Although data on CD47 expression and its correlation with the clinical outcomes of osteosarcoma are limited, we suggest the possibility of CD47 protein expression as a prognostic marker of osteosarcoma. In other cancers, previous studies have demonstrated the association between CD47 protein expression and poor clinicopathological characteristics [28,29,30,31], suggesting the contribution of CD47 in the evasion of cancer cells from immune surveillance. For example, CD47 protein expression has been associated with regional and distant metastases in colorectal carcinoma. For gastric cancer, CD47 protein expression was observed in 57 of 115 patients and served as an independent adverse prognostic factor. In both heterotopic and orthotopic xenograft mouse models, CD47 downregulation significantly suppressed the proliferation and metastasis of cancer cell lines [32,33]. Furthermore, we found that CD47 protein expression was associated with metastasis at the time of osteosarcoma diagnosis. And our in vitro study suggested that CD47 can suppress the migration of osteosarcoma cells. Therefore, CD47 protein expression may serve as a valuable prognostic indicator for osteosarcoma, and targeting CD47 is a promising potential therapeutic approach for this malignancy.

We demonstrated that the CD47 antibody augmented the in vitro phagocytic activity of MDMs towards osteosarcoma cells. Despite the notable success of immune checkpoint inhibitors in various malignancies, achieving satisfactory therapeutic effects in osteosarcoma remains challenging because of the complex tumor microenvironment [34,35]. Furthermore, the immunosuppressive microenvironment within osteosarcomas can impede T-cell activation and function, further contributing to a reduced response to therapy [36]. These challenges highlight the need for alternative immunotherapeutic approaches and innovative strategies to enhance treatment outcomes in patients with osteosarcoma. Recent research has revealed the antigen presentation capabilities of macrophages, extending beyond the conventional roles attributed solely to dendritic cells [37,38]. In various clinical and fundamental investigations, macrophages have emerged as compelling targets for cutting-edge immunotherapeutic interventions. The interaction of CD47 with SIRPα on macrophages is pivotal in this context. Ongoing clinical trials involving CD47 antibodies across various cancer types further underscore the potential of targeting this pathway for innovative immunotherapeutic strategies [39,40,41,42,43]. Macrophages are the predominant immune cell population infiltrating sarcoma tissues, and the evasion of immune surveillance through CD47 signaling may facilitate metastasis. Therefore, CD47 is a potential immunotherapeutic target for osteosarcoma. Our findings provide useful insights for improving treatment outcomes in patients with osteosarcoma.

Our study had some limitations. The inherent rarity of osteosarcoma presents a formidable obstacle to sample acquisition, consequently impeding the practicality of conducting extensive experimentation. The small sample size precluded us from analyzing the association between CD47 protein expression and survival. Due to these limitations, we could not explore the correlation between CD47 expression and metastasis in osteosarcoma biopsy samples in depth. Nevertheless, our cases could represent extreme cases of osteosarcoma, as the proportion of cases with metastasis (25%) was similar to the usual clinical presentation. Our preliminary exploration suggests the promising potential of using CD47 as a novel target for osteosarcoma immunotherapy. Further investigations with larger sample sizes and comprehensive analyses are needed to substantiate and broaden our initial findings. In vivo studies should be performed using a mouse model of osteosarcoma established previously to elucidate the correlation between CD47 expression and metastasis in osteosarcoma cell lines [44]. Furthermore, prospective studies should focus on fortifying phagocytic activity and aligning it with effector T-cell functions, with the ultimate goal of establishing a more nuanced understanding of the potential role of CD47 in osteosarcoma immunotherapy. Despite the limitations of our study, the findings provide valuable groundwork for a better understanding of the role of CD47 in osteosarcoma and suggest an innovative immunotherapeutic approach against this formidable malignancy.

## 5. Conclusions

The association between CD47 protein expression and clinical characteristics in biopsy tissues, along with the enhanced phagocytic activity towards osteosarcoma cells by the CD47 antibody, suggests the potential value of CD47 in osteosarcoma immunotherapy. Despite its limitations, the findings of our study lay the groundwork for understanding the role of CD47 in osteosarcoma and suggest an innovative immunotherapeutic approach against this malignancy.

## Figures and Tables

**Table 2:** Comparison of clinicopathological characteristics based on CD47 positivity.

	CD47-Positive (n = 5)	CD47-Negative (n = 19)	*p*-Value
Age (y)	16.5 ± 6.2	15.7 ± 3.0	0.07
Sex	3 M, 2 F	13 M, 6 F	0.72
Metastasis at diagnosis	3/5 (60.0%)	3/19 (15.8%)	0.04
Tumor length > 8 cm (AJCC T2)	3/5 (60.0%)	10/19 (52.6%)	0.77
Good response to preoperative chemotherapy (<10% residual viable tumor)	2/5 (40.0%)	13/19 (68.4%)	0.24

## Data Availability

Data are contained within the article.
